# Reciprocal perspective as a super learner improves drug-target interaction prediction (MUSDTI)

**DOI:** 10.1038/s41598-022-16493-9

**Published:** 2022-08-02

**Authors:** Kevin Dick, Daniel G. Kyrollos, Eric D. Cosoreanu, Joseph Dooley, Joshua S. Fryer, Shaun M. Gordon, Nikhil Kharbanda, Martin Klamrowski, Patrick N. L. LaCasse, Thomas F. Leung, Muneeb A. Nasir, Chang Qiu, Aisha S. Robinson, Derek Shao, Boyan R. Siromahov, Evening Starlight, Christophe Tran, Christopher Wang, Yu-Kai Yang, James R. Green

**Affiliations:** 1 Department of Systems and Computer Engineering, Carleton University, Ottawa, ON Canada; 2 Institute of Data Science, Carleton University, Ottawa, ON Canada

**Keywords:** Computational models, Machine learning, Target identification

## Abstract

The identification of novel drug-target interactions (DTI) is critical to drug discovery and drug repurposing to address contemporary medical and public health challenges presented by emergent diseases. Historically, computational methods have framed DTI prediction as a binary classification problem (indicating whether or not a drug physically interacts with a given protein target); however, framing the problem instead as a regression-based prediction of the physiochemical binding affinity is more meaningful. With growing databases of experimentally derived drug-target interactions (e.g. Davis, Binding-DB, and Kiba), deep learning-based DTI predictors can be effectively leveraged to achieve state-of-the-art (SOTA) performance. In this work, we formulated a DTI competition as part of the coursework for a senior undergraduate machine learning course and challenged students to generate component DTI models that might surpass SOTA models and effectively combine these component models as part of a meta-model using the Reciprocal Perspective (RP) multi-view learning framework. Following 6 weeks of concerted effort, 28 student-produced component deep-learning DTI models were leveraged in this work to produce a new SOTA RP-DTI model, denoted the Meta Undergraduate Student DTI (MUSDTI) model. Through a series of experiments we demonstrate that (1) RP can considerably improve SOTA DTI prediction, (2) our new double-cold experimental design is more appropriate for emergent DTI challenges, (3) that our novel MUSDTI meta-model outperforms SOTA models, (4) that RP can improve upon individual models as an ensembling method, and finally, (5) RP can be utilized for low computation transfer learning. This work introduces a number of important revelations for the field of DTI prediction and sequence-based, pairwise prediction in general.

## Introduction

The elucidation of drug-target interactions (DTIs) are needed to characterize the physiochemical binding affinity of potential drug compounds to a given protein target. The determination of DTI interactions enable applications such as drug repurposing and screening which are of great importance in light of emergent diseases and viruses as exemplified in the recent and ongoing COVID19 pandemic^1^. The computational identification of novel DTIs accelerates the drug discovery process and enables the rapid discovery of putative interactions representing candidates for experimental validation.

Historically, many computational DTI methods have formulated the problem as a binary classification where predictions between a given drug and protein target are expressed as either binding or not^2–7^. Such a formulation does not necessarily capture the multitudinous continuous-value factors that results in a DTI, such as respective molecular concentrations. When treated instead as a regression-type problem, where the input drug-target pair representation are predicted as a continuous drug-target affinity (DTA) value, the DTI prediction produces a more nuanced representation of relative binding affinity. Many of the contemporary DTI benchmark datasets (namely, BindingDB^8^, Davis^9^, and KIBA^10^) express drug-target binding affinity in a quantitative measure; however, the measures used in each dataset are not necessarily compatible.

Binding affinity is quantified and expressed in various ways. For example, it may be represented as a dissociation constant ($K_d$), or as an inhibition constant ($K_i$), or the half maximal inhibitory concentration ($IC_{50}$). When the $IC_{50}$ value is low, it indicates high binding affinity. Similarly, a low $K_i$ value indicates a high binding affinity. Generally, $K_d$ and $K_i$ values are expressed in terms of $pK_d$ and $pK_i$ respectively, which stand for the negative logarithm of $K_d$ and $K_i$.

The research community has focused on the training and testing of deep machine learning DTI methods on independent datasets with their uniquely expressed definitions of binding affinity. Three datasets have formed the benchmark basis for the development of SOTA DTI methods. The Davis and BindingDB, while smaller than the KIBA dataset, each express DTI pairs using a traditional DTI binding affinity ($K_d$) that, for consistency, we will refer to as $K_d$ in this work^8,9^. Complimentarily, the KIBA dataset defines DTI binding affinity using an aggregate ‘KIBA Score’ (we denote it as $K_s$ in this work) that combines several DTI metrics into a single meta-score^10^. While the $K_d$ value of certain DTI pairs contribute to the overall $K_s$ score, numerically, there is no simple, linear mapping between the two metrics.

### The combination of benchmark datasets for double-cold evaluation

Given the importance of these three benchmark datasets to DTI prediction, we here define a new experimental method to integrate the three benchmark datasets such that they might be leveraged to train new models on the maximal number of available pairs to advance the DTI SOTA, while providing a framework for fair comparison with existing methods. To that end, we focus on the development of models trained on the combined BindingBD and Davis datasets (given their consistent definition of DTI scoring with binding affinity, $K_d$) and then independently evaluate performance over the larger KIBA dataset. Most importantly, in considering all three datasets, we additionally define a novel evaluation dataset, denoted the ‘double-cold dataset’, where no one drug SMILE or protein amino acid sequence appears in either the training or validation datasets. Since this final evaluation dataset is completely independent of the training and validation datasets, it correctly reflects model performance when applied to completely new drug SMILES and/or completely new proteins. Consequently, it is an evaluation framework that better represents how performant various SOTA models would be in generating predictions for new emergent diseases (such as is the case for emergent diseases like COVID-19 caused by the SARS-CoV-2 proteins).

In an effort to formalize this research project as part of a collective project tailored to senior-undergraduate students to generate models that might surpass SOTA DTI models, we challenged a cohort of students to generate deep machine learning models that may surpass SOTA DTI models. In the following sections, we lay the conceptual foundations that encouraged the generation of novel DTI SOTA models. This initiative was predominantly inspired from global-centric challenges seen within distributed competitive frameworks. In the following sections, we describe how the regression-based formulation of the DTI task within a peer vs. peer challenge framework enabled the generation of competitive SOTA DTI models.

### Computational competition breeds innovation

The big data and artificial intelligence era has enabled the establishment of computational variants of traditional fields of research (and their pedagogical frameworks) including computational biology^11^, computational chemistry^12^, and computational physics^13^. However, it is within global and/or community-level competition contexts that many state-of-the-art (SOTA) methods emerged. The framing of grand challenges in ways that engage the broad research community enables the consistent use of benchmark data, the cross-pollination of methodologies, and progressive iteration of achievable performance^14^. In the following subsections, we describe how computational competitive contexts enabled this work.

First, we introduce the Netflix Competition as it arguably represents the initial demonstration of galvanizing both academic and industry research groups in the pursuit of a large-scale and multi-year challenge. Secondly, the success of the Netflix Competition gave rise to the Kaggle online competitive framework. Finally, we describe how these frameworks are increasingly used as the basis of grand challenge competitions within biomedical research to advance the frontier of domain knowledge.

#### The Netflix competition

While specific academic research communities enjoyed the burgeoning of computational compliments over the lat decade(s), it is the nascence of the Netflix competition circa. 2006–2009 that initiated the research community and global community to a benchmark-based innovation challenge. In 2006, Netflix publicly released a dataset comprising a hundred million anonymized movie ratings on a five-point scale as part of a million-dollar challenge to the global computer science and machine learning communities to beat its existing recommendation system, denoted Cinematch^15^.

The Netflix competition represents one of the original framings of an international competition soliciting the efforts of teams of researchers applying themselves to improve (at the time, by a substantial margin) the state-of-the-art method for a specific task. In providing a high-quality and structured dataset from which teams could base their solutions^15^, numerous advances in the research of recommendation systems were achieved^16–19^.

Most notably, the top-ranking competitors incrementally generated large ensembled methods from individual component predictors; the best performing models resulted from the combination of complimentary methods and from multi-scale views^14^. These large-scale ensemble methods integrated through a cascaded linear model are typically referred to as a “blended” model in machine learning literature^20^. Through the use of *k*-fold cross-validation for creating a weighted combination of many candidate learners, these ensemble models are referred to as “super learners”^21^. The last decade has increasingly seen the use of ensemble methods as part of online competitions since the completion of the Netflix Competition. The official winners of the multi-phasic competition (under the pseudonym “BellKor Pragmatic Chaos”) achieved the ambitious minimum Root Mean Squared (RMSE) improvement of +10% over the Cinematch and other baseline solutions on September 18, 2009^22^. The popularization of this competition gave rise to a trend that has since shaped the landscape of crowd-sourced solutions to otherwise challenging open industrial and research questions.

#### Kaggle: online competition environment

Through the decade following the Netflix competition (2010–2020), a paradigm shift in the crowd-sourced problem-solving space ensued. Open innovation and crowd-sourcing organizations, such as InnoCentive, offered monetary rewards to selected “Solvers” for proposed solutions to posted “Challenges” of unsolved problems^23^. However, it is the fully online competition frameworks, such as Kaggle, that fostered communities of machine learning practitioners and data scientists to crowd-source solutions to open problems^24^. The Kaggle platform enables users to leverage published datasets, contribute models, and collaborate broadly to solve machine learning/data science problems^24^. The crowdsourcing of solutions be collaborative and/or competitive depending on the challenge outcome. A collaborative challenge seeks to focus contributors towards an objective outcome that is achieved incrementally and rewards shared contribution. A strictly competitive problem formulation (e.g. InnoCentive) often seeks to collect diverse and independently generated solutions from which the crowdsourcer selects a winner^24^.

Furthermore, the platform enables individuals to establish credentials on open datasets in a structured environment. This formal extra-university framework for establishing one’s expertise in machine learning represents a new form of credentialization that can lead to employment opportunities both within the Kaggle ecosystem and beyond. Much like the Netflix competition, submitted solutions are automatically evaluated against a benchmark enabling the ranking of teams in near-real-time.

In essence, contemporary problems and corresponding structured datasets are made broadly available to the global community during a competition period and top-ranking solutions receive monetary prizes for their solutions. As an online collaborative environment, participants are also rewarded for contributing open programmatic content that is up-/down-voted by other participants based upon its utility to the community. In summary, Kaggle has fundamentally transformed the data science and applied machine learning landscape through democratization of datasets and methods in a fully collaborative digital environment available to all, expert to student alike, in contrast to the global competitions directed by dedicated research communities to tackle fundamental research questions.

#### Critical assessment of <Insert Task>

With a more dedicated research focus, international competitions to address grand challenges are often run at biennial intervals. For example, in the pursuit of advances at the frontier of molecular biology, a series of competitions templated by the convention “Critical Assessment of <Insert Task>” are hosted to encourage the development of new methods and derive novels insight towards the resolution of each challenge^25^. Examples include the “Critical Assessment of Structure Prediction” (CASP; now in its 15th iteration)^26^, the “Critical Assessment of Genome Interpretation” (CAGI; now in its 6th iteration)^27^, and the “Critical Assessment of Functional Annotation” (CAFA; now in its 4th iteration)^28^ competitions, among others. CASP was the first of such competitions after which other fields modelled themselves. These international competitions are held regularly to galvanize teams within the research community to develop methods in an effort to establish and advance the state-of-the-art. Similar to the Kaggle challenges, these competitions provide research groups with an opportunity to establish their excellence in a fair and open competition, with some groups participating to solve the challenge, while others participate to establish the superiority of their underlying machine learning methods.

Teams are not exclusive to academic research labs; in a notable example, during the 2020 CASP competition, DeepMind developed AlphaFold resulting in a tremendous improvement in performance over competing methods and benchmarked across previous years^29^. The competition assessors declared that AlphaFold 2 had succeeded in solving the 50-year grand challenge demonstrating the promise of machine learning when used in conjunction with massive-scale computational resources^30^.

As with Kaggle competitions, these international competitions represent excellent learning opportunities for participating students (whether at the graduate or undergraduate level) to establish credibility in the sub-field, or more broadly, within machine learning. (Under)graduate student-based teams with the guidance of a seasoned mentor can, at times, be successful within these competitions.

### MetaStudent: a student-centric case study

Competitions, whether tailored to research or industry applications, represent excellent learning opportunities and, consequently, may be leveraged for pedagogical goals. In an impressive demonstration of utilizing graduate student bioinformatic pedagogy for the advancement of research in the domain of protein function prediction, as part of a 2013 Master’s-level bioinformatics course, Dr. Burkhard Rost (Technical University of Munich) assigned the project of generating solutions for the recently-run CAFA competition^31^. The 16 students participating in the course were divided into three groups and each applied themselves to generating a homology-based model capable of predicting protein functions, resulting in three solutions denoted “StudentA”, “StudentB”, and “StudentC”. Two of these three methods were determined to be competitive in CAFA and outperformed related methods prompting their combination as part of a single meta-predictor^31^.

The post-CAFA evaluation of the meta-model (denoted *MetaStudent* as a weighted ensemble based on confidence scores of each model) was evaluated to have been among the top-10 methods of the competition; a notable feat for student-only teams producing their models over an 8-week period^31^. This work represents a promising demonstration that tailoring student pedagogy to include projects focused on addressing open research questions may lead to surprisingly successful outcomes that contribute to the research community well beyond the ephemerality of that course^32^; MetaStudent, to date, is incorporated in the PredictProtein software developed by the Rost lab^33^.

This work inspired the generalized framework presented in this article to tailor the pedagogy of a senior undergraduate coursework to address open research questions. The following sections describe how this was achieved to support the global COVID19 pandemic research initiative to encourage students to contribute meaningfully to contemporary problems, as outlined in^32^.

### Related work and ensembling student-generated DTI methods

The related DTI work covers a broad collection of methods as summarized in recent reviews^34^. Recent (deep) machine learning approaches have formulated the drug-target prediction problem so as to predict the continuous DTA value directly.

One of the first such methods was proposed by Pahikkala et al. that combined a drug compound similarity-based representation and Smith-Waterman similarity representation of targets in conjunction with the Kronecker regularized least-squared method^35^. The work of Zhao et al. introduced a DTA model based on an adversarial neural network (GAN) architechture^36^, denoted GANsDTA, capable of learning drug and protein sequence features for both labelled and unlabelled data^37^. In the work of Abbasi et al., denoted DeepCDA, the combined use of convolutional neural networks (CNNs) and long-short-term memory (LSTM) blocks produced a two-sided attention mechanism that learns a better representation of drug and protein sequences^38^. In the work of Öztürk et al., describing the SOTA DeepDTA model, a one dimensional CNN encoding of the drug SMILES and proteins sequences are concatenated into a single representation and fed in multiple deep fully connected layers to produce a final output prediction^39^. Shim et al. proposes a similarity-based model that generates DTA predictions from two dimensional CNNs applied to the outer products between column vectors of two similarity matrices for the drugs and targets^40^.

Beyond the use of a single (deep) machine learning model for DTI prediction tasks, there is additionally a growing usage of an ensembled collection of individual models to achieve and/or surpass SOTA-level performance. For example, the EnsembleDLM method embeds sequence information of chemical compounds and proteins and generates predictions based on the aggregation of predictions from multiple deep neural networks; this method achieved SOTA performance over the Davis and KIBA datasets^41^.

Most relevant to the work presented herein is the concept of Super Learners^21^ that integrate the predicted output of numerous individual component learning models within a cascaded learning model that generates a final prediction. Super learners are conventionally trained through *k*-fold cross-validation and benefit from the complimentary input of numerous diversely-defined component predictors. Most importantly, their training, validation, and test datasets are specifically defined to guarantee that no training sample is present in the test sets to ensure a fair comparison and integration of methods.

**Figure 1:**
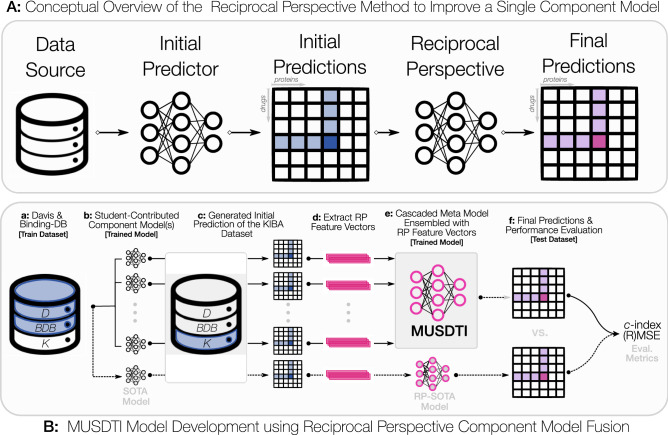
Conceptual overview of the proposed MUSDTI predictor.

In Fig. 1 we illustrate how the three DTI benchmark datasets were leveraged to enable the training and evaluation of student-contributed deep machine learning DTI models and how these were combined using the Reciprocal Perspective (RP)^42,43^ framework to form a meta-DTI model that was evaluated against SOTA DTI models. In combining multiple component student models as part of an ensembled meta-model, we demonstrate that significant improvement in performance over SOTA models can be achieved and the experimental design employed should serve as the basis for future DTI model development to adequately report expected model performance on double-cold DTI pairs (where neither the drug SMILES nor target amino acid sequence have previously been seen in training or validation data).

## Data and methods

In the following section, we describe how three of the benchmark DTI datasets were integrated into training, validation, and the proposed double-cold evaluation dataset. The training data were then made available to students as part of an senior undergraduate machine learning course project. We describe the software framework, programming environment, and hardware available to the students. We then describe how each of these student contributed component models were combined using the RP cascaded machine learning method and finally evaluated and compared to SOTA DTI methods on the double-cold dataset as well as the test dataset defined in the DeepDTA work, for a fair comparison^39^. An overview figure of the end-to-end processing pipeline is visualized in Fig. 1.

### Structured project materials for both didactics and contributing to contemporary research

Students enrolled in the Fall 2020 course offering of Introduction to Machine Learning at Carleton University (SYSC4906) were instructed as part of their course-long project to produce machine learning models competitive with SOTA DTI models^32^. To that end, students formed groups of two and were provided with the BindingDB and Davis datasets as well as Jupyter Notebooks utilizing the DeepPurpose framework^44^ demonstrating how a DTI model could be trained and evaluated.

To ensure all students shared equal access to hardware resources with which to train and evaluate their models, Google’s Collaboratory environment (Google Colab) was used by each student group. The adoption of notebook environments and freely accessible cloud-based high-performance computing infrastructure for scientific discovery is emergent and democratizing the scientific process^45^. While access to high-capacity GPUs varies by session, anecdotally, all student groups were able to train and evaluate multiple iterations of their proposed component DTI models over the project duration. A discussion of the many lessons learned in formulating an open research project as part of (under)graduate course didactics are available in our related work^32^. The following sections detail the dataset, software framework, RP methodology, and experimental design with a comparison against SOTA DTI methods (Fig. 1).

### The Davis, BindingDB, and KIBA datasets

**Figure 2:**
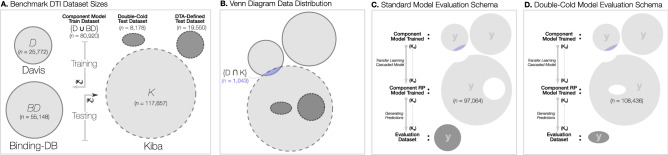
Experimental design to evaluate the proposed MUSDTI predictor.

In this work we leverage three benchmark datasets to train and evaluate the component student models. The smallest of the three, Davis, includes binding affinities (expressed in $K_d$) for $\sim$26,000 pairs involving 68 unique drugs and 442 unique targets^9^. The second database, BindingDB, is a a publicly accessible database of experimentally measured binding affinities for $\sim$55,000 pairs (as of time of writing,Feb. 6, 2022, the dataset now contains 41,296 unique entries representing 8661 protein targets and 1,039,940 small molecules)^8^. Similar to the Davis dataset, the recorded binding affinities are expressed as a $K_d$ value. Finally, the most unique benchmark dataset of the three is KIBA that integrates kinase inhibitor bioactivity from various affinity measurements including $K_d$, $K_i$, and $IC_{50}$ in a uniquely defined “KIBA score”, denoted as $K_s$ in this work^46^. The KIBA dataset comprises $\sim$118,000 observations (involving 52,498 drugs and 467 targets). Table 1 summarizes the sizes of each dataset for use in each stage of the model generation pipeline illustrated in Fig. 1 and according to the experimental design outlined in Fig. 2. Finally, while prior work may have considered the direct $K_d$ values from Davis or BindingDB^35^, we followed from the work of Öztürk et al.^39^, He et al.^46^, and as implemented in Shim et al.^40^ to log-transform the values into a $pK_d$ value as follows:

1 $$\begin{aligned} pK_d = -\log _{10}\Big (\frac{K_d}{1e^{9}}\Big ) \end{aligned}$$

Importantly, we note that a linear mapping between $K_d$ as used in the Davis and BindingDB datasets and the $K_s$ used in the KIBA dataset does not exist. In the majority of DTI model development, methods are trained and evaluated on independent datasets. Promisingly, the public availability of these three benchmark datasets in developing DTI predictors (in a safe and efficient way) within this work may ultimately lead to improved DTI SOTA models. To that end, students were tasked with producing a DTI regression model trained from both the Davis and BindingDB datasets to then leverage the KIBA dataset. Subsequently, their models were further refined and improved using a cascaded RP model. As previously described, students were provided with access to these two benchmark datasets and the ability to recreate and evaluate existing SOTA DTI models through the DeepPurpose framework which, ultimately, enabled them to generate their own custom DTI models.

**Table 1:** DTI dataset sizes and their combined usage in defining the two test datasets.

Dataset descriptor	Num. DTI pairs
Davis	25,772
BindingDB	55,148
KIBA	117,657
Training data (D+BDB)	80,920
Numerical Map data (KIBA)	108,436
Test size (double cold)	8178
Test size (DTA-defined)	19,550

### The DeepPurpose framework

Several models produced with the DeepPurpose framework were compared with KronRLS^35^, and GraphDTA^47^, and DeepDTA^7^, each state-of-the-art DTI methods. It was concluded that some methods using DeepPurpose achieved comparable predictive performance on two benchmark datasets, DAVIS^9^ and KIBA^10^; this work integrates multiple benchmark datasets and fairly investigates how the DeepPurpose framework enables the extension and integration of these studies. The DeepPurpose framework, as originally introduced by Huang et al. in the Fall of 2020^44^ is a deep learning-based molecular modeling and prediction toolkit that provides a programmatic framework enabling the rapid prototypting of DTI predictors and related molecular computational applications including protein function prediction, protein-protein interaction prediction, and compound property prediction^44^. In abstracting away much of the low-level programming required to load, process, and manipulate drug SMILES and protein amino acid sequences, DeepPurpose makes readily accessible the implementation of seven protein encoders, eight compound encoders, over 50 deep learning models. Huang et al. empirically determined that models implemented in DeepPurpose and evaluated against SOTA DTI predictors achieved similar or improved performance on DTI benchmark datasets^44^. Promisingly, the DeepPurpose documentation highlights among its features that numerous combinations of drug-target encoding and deep learning models have yet to be trained and evaluated, leaving considerable room for individual and ensembled models to be produced:

“15+ powerful encodings for drugs and proteins, ranging from deep neural network on classic cheminformatics fingerprints, CNN, transformers to message passing graph neural network, with 50+ combined models! Most of the combinations of the encodings are not yet in existing works. All of these under 10 lines but with lots of flexibility! Switching encoding is as simple as changing the encoding names!”

To that end, the approximately 39 student teams in the course were introduced to DTI prediction and provided notebooks using the DeepPurpose framework to implement and retrain an existing SOTA model, notably the DeepDTA model by Öztürk et al.^39^ and then challenged to produce their own model in an attempt to improve performance over this existing model, replicating a Kaggle-like competition.

### Development and comparison of component models

Instructed only to make use of either or both the Davis and BindingDB datasets to develop their models, students trained their component models (CM) using the DeepPurpose framework and Google Colab for access to GPU resources. For the small number of duplicate pairs in the two training datasets ($n=1043$) the label was set to the average of those scores. We distinguish individual student models as “component models” to differentiate them from subsequent ensemble models. Each component model was assigned a unique identification templated as G<id> where <id> $\in \{1,2,3 \ldots , 39\}$. With this naming convention, we refer to the component model produced by group 9 and “G9-CM”.

In Fig. 2A we illustrate the relative sizes of each of the three datasets considered in this work and emphasize their binding affinity measure (i.e. $K_d$ for training, $K_s$ for testing); a Venn diagram of how each dataset overlaps and relates to the others is visualized, noting that the two test datasets (the ’double-cold’ and ’DTA-defined’) are subsets of KIBA, as illustrated in Fig. 2.

For a fair comparison against the DeepDTA SOTA DTI model, we considered the same evaluation dataset as defined in Öztürk et al.^39^ that we denote as the “DTA-Defined Test Dataset” in Fig. 2C. The second test dataset is a considerably more challenging “Double-Cold Test Dataset” given that it comprises the set of pairs where neither the drug compound or target protein appear in the either thge BindingDB $\cup$ Davis training dataset (Fig. 2D). Consequently, this dataset represents the most challenging evaluation task and reflects the model performance when predicting completely novel and/or unseen drug targets and drug compounds. For novel organisms or emergent pathogens/viruses, top-performing models evaluated under this proposed scheme are ideally suited.

### Reciprocal perspective for transfer learning over KIBA to generate RP-DTI models

As described above, there is no simple linear mapping between $K_d$ and $K_s$, such that the three benchmark datasets cannot be easily combined for model training and evaluation. We hypothesized that a cascaded model could be trained to learn the nonlinear mapping between these DTA definitions.

**Figure 3:**
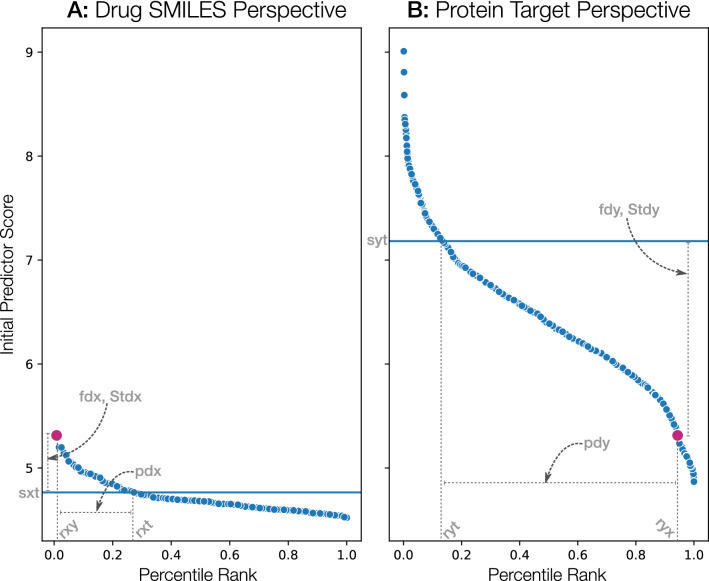
Example paired one-to-all score curves. An example pair demonstrating dramatically differing distributions is depicted to emphasize that even though a given drug scores relatively low in the given protein target perspective, that protein is the top-scoring target for that specific drug.

For similar bioinformatic tasks, the Reciprocal Perspective (RP) framework has been leveraged in a cascade to improve initial model prediction results^42,43^. In subsequent work, RP was used for the cascaded combination of multiple experts ($n = 2$)^48^, and we here hypothesize that RP can be used not only for the combination of multiple experts ($n \gg 2$) but also as a means of learning a domain transfer model. We depict in Fig. 3 the various RP-based features extracted for any pair of drug and protein target.

Leveraging all three benchmark datasets with two differing DTA measures connected through a non-linear mapping function provides the opportunity to use the RP framework to generate a cascaded learning model to learn this non-linear mapping and further improve the component model performance. In previous work, RP was demonstrated to significantly improve the predictive performance of protein-protein interaction predictors^42^ and the performance of microRNA target predictors^43^. RP has also been shown capable of fusing component models ($n=2$) as an ensembling method^48^. In this work, we propose to utilize RP as both a transfer learning method (to learn an approximated mapping from $K_d$ to $K_s$) as well as a many-CM ensembling method ($n\gg 2$).

As illustrated in Fig. 1, the prototypical prediction pipeline will consider a given data source to train and evaluate a model and generate a set predictions, however RP cascades beyond these initial results. The RP method makes use of the comprehensive set of all predicted scores (denoted the “Comprehensive Prediction Matrix” [CPM]) as a data source (i.e. in a cascade) to train and evaluate a downstream RP model to generate the final set of predictions^42^.

This methodological framework was originally introduced as a cascaded, semi-supervised learning algorithm to improve the pair-wise predictive performance of existing learning algorithms. Most interestingly, in considering these output scores generated by various initial learning algorithms as a combined input to the RP method, those model-specific scores are cast into a new rank-order domain denoted a One-to-All score curve (O2A) where, in the case of DTI provides two complimentary views, a drug-based “perspective” and a protein-based “perspective”. That is, for an *n*-numbered set of drugs and *m*-numbered proteins, the CPM containing all nm/2 predictions could be utilized through RP. For a given query pair (*x*, *y*), RP examines the pair’s predicted score in the context of all predicted scores for all pairs involving either (*x*,$*$) or ($*$,*y*). The RP method differentiates itself from other cascaded predictors in that it is domain-agnostic (the features leveraged are derived from a domain entirely independent of the context of the original problem) and it therefore serves as a prediction error-correction layer.

Intuitively, it is the recent development of high-throughput (deep) learning models that have enabled the generation of the comprehensive scoring of all possible pairs of elements (i.e., CPM). This has given rise to context where we can appraise the relative value of one element with respect to all others (e.g., how does the score of one potential protein target compare to the scores of all possible targets?). RP extends this further by examining each perspective reciprocally (e.g., in-context score of the target and in-context score of the drug), such that the score of the pair can be placed in context. The RP framework estimates from all predicted scores a localized baseline on a per-element basis (e.g. per-drug and/or per-protein) enabling the computation of a number of rank-order metrics.

By considering a putative interaction of elements from the perspective of each of the elements within the pair, the RP framework extracts 14 pair-specific features (tabulated in Table 2) as input features to train a cascaded super learner model. These features contextualise each pair among all other predicted pairs thus making use of semi-supervised distributions and a variety of features types (including rank, fold, statistics, and score-types). Thus RP rescores the predicted DTI pair as part of a cascaded super learning model, typically an eXtreme Gradient Boosting (XGBoost) model^49^ as in Kyrollos et al. and Dick et al.^43,48^.

**Table 2:** The 14 RP features derived from DTI pair-specific one-to-all score curves.

Feature generic name	Short name	Type	Description
Y-in-X-percentile	*ryx*	Rank	Percentile of target Y among all the predictions for drug X
X-in-Y-percentile	*rxy*	Rank	Percentile of drug X among all the predictions for target Y
Adjusted reciprocal rank order	ARRO	Rank	Reciprocal product of *rxy* and *ryx*
X-percentile-baseline	*rxt*	Rank	Percentile rank of the target nearest to the local cutoff value of drug X
X-baseline	*sxt*	Score	Score at the local cutoff value of drug X
Y-percentile-baseline	*ryt*	Rank	Percentile rank of the drug nearest to the local cutoff value of target Y
Y-baseline	*syt*	Score	Score at the local cutoff value of target Y
Percentile-difference-from-baseline-X	*pdx*	Fold	Difference between *rxy* and *rxt*
Percentile-difference-from- baseline-Y	*pdy*	Fold	Difference between *ryx* and *ryt*
Fold-difference-from-baseline-X	*fdx*	Fold	Fold-difference of target Y score in drug X from baseline *sxt*
Fold-difference-from-baseline-Y	*fdy*	Fold	Fold-difference of drug X score in target Y from baseline *syt*
SD-distance-from-mean-X	*Stdx*	Stats	The number of standard deviations from the mean score in drug X
SD-distance-from-mean-Y	* Stdy*	Stats	The number of standard deviations from the mean score in target Y
Original-Score	$G<id>$	Score	The original predicted score from the component model

To generate the cascaded RP model for each student-generated CM, each CM was used to generate the comprehensive predictions of all n(n+1)/ 2 drug-target pairs of the KIBA dataset, denoted the CM comprehensive prediction matrix (CPM), and thereby producing a predicted $K_d$ affinity score for each pair (originally expressed as $K_s$ when available). This large-scale prediction task made use of the high-performance compute infrastructure provided by Compute Canada to massively parallelize the prediction generation over KIBA. For the benefit of the broader research community, we release the complete set of our CM predictions over KIBA for reproducibility and from which subsequent projects might benefit (discussed in the Future Directions below).

Once predicted, the 14 RP features for each component model as defined in Table 2 were computed and a cascaded RP XGBoost model (with hyperparameters defined in Table 3) for each CM was trained. We optimized, through grid-search, five particular hyperparameters in the in the training of the MUSDTI and the MUSDTI* contributing models (*colsample by tree*, *gamma*, *learning rate*, *max depth*, and *minimum child weight*); these values were determined through the use of the validation sets available to each model.

**Table 3:** MUSDTI hyperparameter values.

MUSDTI model parameter	DeepDTA dataset	DeepDTA* dataset	Double-cold dataset	Double-cold* dataset
Colsample by tree	0.9362	0.7991	0.8988	0.9278
Gamma	1.1306	2.918	1.9723	4.342
Learning rate	0.2637	0.095	1.748	0.093
Max depth	15.0	13.0	12.0	12.0
Min child weight	2.0	9.000	4.0	1.0

The * designation denotes the model parameters used for the ensembled MUSDTI* model prior to the cascaded application of RP.

To differentiate the performance attributable to the application of the context-leveraging RP to each component model versus the performance attributable to the numerical mapping procedure alone (mapping $K_d$ to $K_s$), we also trained CM-specific numerical mapping (NM) models. The NM models were trained using a single feature, the predicted $K_d$ of a KIBA pair, with the goal of effectively learning the non-linear mapping between $K_d$ and $K_s$. The numerical mapping models are a simple XGBoost model (with default hyperparameters) used to learn the non-linear mapping between two numerical domains to effectively “translate” $K_d$ values to $K_s$ values. This non-linear mapping procedure is expected to be learned by the cascaded MUSDTI meta-model, however, to fairly compare the performance of the meta-model with the component models, they must each be evaluated based on the output score generated for the evaluation dataset. Consequently, the CM-NM models are produced using the same meta-model training samples however only take as input the CM-generated $K_d$ score and learns to map it to the ground-truth $K_s$ value. The application of RP to each CM (the CM-RP models) leverages all 14 RP features.

Finally, to demonstrate RP as a many-CM ensembling method with the goal of producing the best performing model (a super learner), we iteratively fused the RP-features of all CMs and trained new multi-CM-RP models, re-evaluating the performance of each. We selected the order of progressively included models based on the rank-order performance of each CM over the validation dataset. This work sought to determine the trade-off between the performance increase from progressively including CM and the computational expense in adding each. The inference rate for each model was therefore reported to express the relative runtime as an inference rate (i.e. number of predicted pairs per unit of time).

All models were fairly evaluated over the two test datasets where we considered two complimentary performance metrics; the first focused on the agreement between prediction and ground-truth, and the second on the ordering of predictions. The first evaluation metric considered is the Root Mean Squared Error (RMSE) defined as:

2 $$\begin{aligned} \text {RMSE}=\sqrt{\frac{1}{N}\sum ^{N}_{i=1}(y_{i} - \hat{y_{i}})^2} \end{aligned}$$

where $\hat{y_i}$ is the predicted value and $y_i$ is the ground-truth value. Smaller RMSE values represent better models, and vice versa.

Given that this work considers multiple datasets with differing metrics expressing DTA, the second magnitude-independent evaluation metric considered is the Concordance Index (CI), or *c*-score. In considering this complementary metric, we could better evaluate the specific influence of the numerical mapping and RP performance contributions. The CI is defined as the proportion of concordant pairs divided by the total number of possible evaluation pairs. Intuitively, the CI focuses on the order of the predictions rather than the magnitude of the predictions themselves. Specifically, the CI over a set of paired data expresses the probability that the predictions for two randomly drawn drug-target pairs with different labels are in the correct order, that is, that the prediction $\hat{y}_i$ for the larger affinity $y_i$ is larger than the prediction $\hat{y}_j$ for the smaller affinity value $y_j$. Formally:

3 $$\begin{aligned} \text {CI}=\frac{1}{Z} \sum ^{}_{y_{i} > y_{j}}h(\hat{y}_{i} - \hat{y}_{j}) \end{aligned}$$

where *Z* represents a normalization constant and *h*(*x*) represents the step function:

4 $$\begin{aligned} h(x)= {\left\{ \begin{array}{ll} 1, &{} \text {if } \begin{aligned} x > 0\\ \end{aligned}\\ 0.5, &{} \text {if } \begin{aligned} x = 0\\ \end{aligned} \\ 0, &{} \text {if } \begin{aligned} x < 0\\ \end{aligned} \end{array}\right. } \end{aligned}$$

The CI ranges between 0.5 and 1.0, where 0.5 corresponds to a random predictor and 1.0 corresponds to perfect prediction accuracy, thus larger CI values represent better models.

### Comparison with SOTA DTI methods

Finally, in order to fairly compare our proposed methods to the SOTA DTI methods, we recreated the implementation of the DeepDTA by Öztürk et al.^39^. This ensured that our proposed model(s) and DeepDTA had accesses to the same information through all stages of the evaluation pipeline enabling direct comparison of our results. Moreover, the DeepDTA model could also be evaluated with a cascade NM and RP layer to demonstrate improved performance resulting from these cascaded approaches.

The DeepDTA model architecture comprises two independent CNN blocks (encoders) to learn a drug SMILES representation and a protein amino acid sequence representation. Both encoders are composed of three consecutive 1D convolutional layers that feed into a max-pooling layer. The two CNN encoder outputs are then concatenated into a single vector that is passed into three fully connected layers, before ultimately producing the output affinity prediction.

In this work, we consider three variants of the DeepDTA model, denoted DTA-BD, DTA-D, and DTA-DBD to represent whether the model was originally trained on the BindingDB dataset, Davis dataset, and joint BindingDB & Davis datasets, respectively. Each of these SOTA models, much as with the CMs, were treated independently of each other through the prediction pipeline (that is, each model was trained on either BindingDB and or the Davis datasets). For each of the experiments considered in this work the three DeepDTA-$*$ models are ranked among all CMs and visually depicted differently to emphasize their performance in relation to the student CMs.

## Results and discussion

In this work, we propose several adaptations of the experimental design for producing and evaluating DTI predictors. Building upon recent deep learning advances for DTI prediction, we propose a novel evaluation framework that makes use of three commonly used benchmark DTI datasets to maximally utilize the available DTI data and incorporate a cascaded transfer learning layer to accommodate the use of differing measurements of binding affinity (i.e. $K_d$ & $K_s$). Consequently, the use of a cascaded learning algorithm (such as RP) to not only learn the $K_d \rightarrow K_s$ non-linear mapping (i.e. numerical mapping models), but to leverage the context enabled by transfer learning these DTA measures (i.e. CM-RP), promises exciting results given that the application of RP to related bioinformatic problems has led to statistically significant improvements of predictor performance^42,43,48^.

In formulating this project as part of senior undergraduate course pedagogy to replicate similar competitive programmatic environments to solve open or active problems, student groups generated individually unique solutions (Supplementary Materials, Table 1), each amenable to evaluation against one another and a fair SOTA DTI prediction model. Conveniently, this formulation also enabled the strict assignment of KIBA data (given it’s relatively large and non-leverageble size) as part of data for use in a cascaded framework. With limited compute infrastucture, students were unable to use large-scale datasets. Given this experimental assignment of DTI pairs, our experimental design could assign the union of Davis and BindingDB pairs as a component model training set, and then define the independent KIBA dataset for the transfer learning task with specifically withheld pairs to represent the test dataset (either as a predefined DeepDTA hold-out test, or the highly conservative double-cold subset).

### Student-generated DTI models outperform SOTA DTI models

Excitingly, in providing students with SOTA DTI models as a starting point in their own development of novel DTI predictors, they had a definitive baseline upon which they might inform their own model development and ultimately compare themselves. As described in Dick et al.^32^, establishing the project baseline as an existing SOTA DTI model spurred innovations to advance the frontier of knowledge.

**Figure 4:**
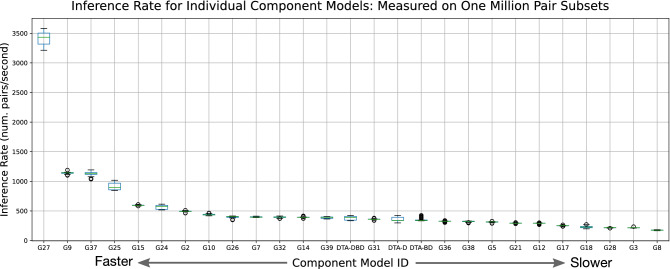
Inference rates of each component model measured over random subsets of 1 million pairs.

Ultimately, student-submitted CM models outperformed the three DeepDTA-$*$ models as reported in Tables 4 and 5. Excitingly, a number of of these student-contributed models also generate predictions on considerably faster time-frames than SOTA models as depicted in Fig. 4; models G27, G9, G37, and G25 all appear to produce predictions faster than all other models that perform similarly.

**Table 4:**
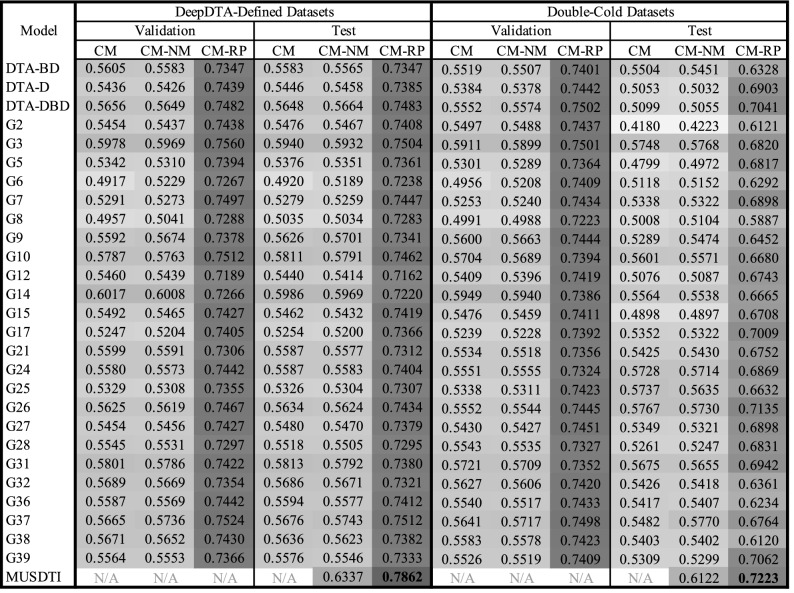
Component and MUSDTI model performance evaluated over the validation and test datasets using concordance index.

**Table 5:**
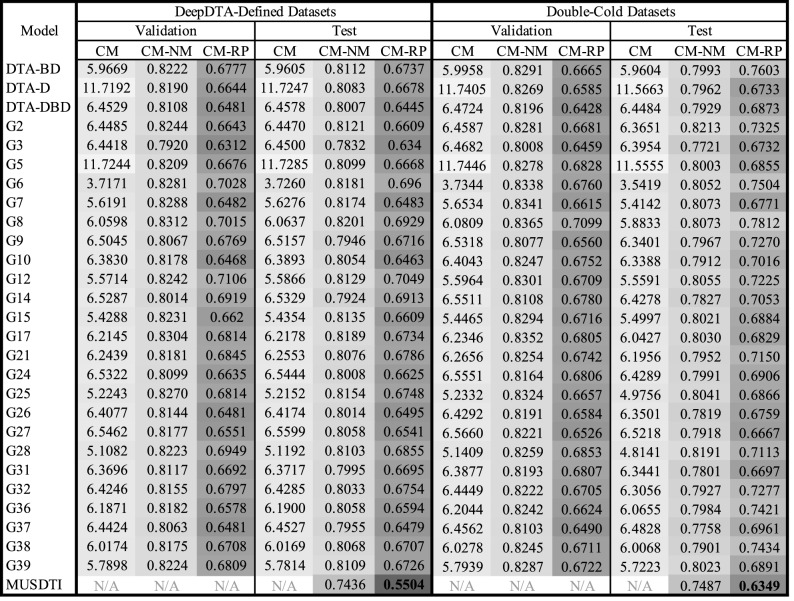
Component and MUSDTI model performance evaluated over the validation and test datasets using root mean squared error.

In considering the reported CM performance of the validation and test sets for the DTA-defined and double-cold tests sets, numerous student CM models outperformed the DeepDTA-$*$ models (Tables 4, 5). For example, in Table 4 summarizing CI results (higher values are better) the highest DeepDTA-$*$ model performance in the CM column is 0.5656 for DTA-DBD (as expected) while several other student-contributed CM models achieve G3:0.5978, G10:0.5787, G14:0.6017, G31:0.5801, amongst others (Table 4).

Similarly, the results measured by RMSE over each of the test datasets corroborate the previous findings: numerous student-contributed findings achieve a smaller RMSE over the DeepDTA-$*$ models. However, given that the reported RMSE is obtained from the $K_d$ prediction of a $K_s$ ground-truth value, these values with differing DTA definition are not meaningful. Rather, the numerically-mapped RMSE values (i.e. CM-NM & CM-RP) are of greater significance (Table 4).

We note that the student-defined CM models effectively represent a large-scale search of hyperparameter space including variable protein amino acid and drug SMILES sequence encodings, variable fusion of single or multiple models, and optimized hyperparameter values according to specific training strategies. Collectively, the exploration of these parameters allo us to draw a various of conclusions based on the selected parameters. Foremost, we note that 9/28 (approximately one third) of student models chose to utilize the same protein and drug 1-D CNN encodings as in the DeepDTA model, however, each group opted to vary other aspects of their prediction pipeline. One notable example, the G3 model, leveraged a fusion-based prediction framework integrating multiple encoding paradigms as part of their methods (differing from the CNN-only approach proposed by Öztürk et al.) and this model consistently demonstrated one of the highest performances amongst the CMs over both datasets. This finding is consistent with the generally known utility of integrating a fusion of multiple complimentary data representations to train a given learning algorithm. Excitingly, various insights can be drawn based on the wide-scale overview of this explored feature space and, to that end, we list the complete CM implemetation details in Supplementary Table 1 and performance results in Tables 4 and 5.

### Learning a numerical-mapper is sufficient for domain transfer

The three benchmark datasets considered in this work expressed DTA in either $K_d$ (BindingDB & Davis) or $K_s$ (KIBA), and to integrate all three in a combined end-to-end framework requires a non-linear mapping to express predicted values in the appropriate domain and can be learned through an additional learned machine learning layer. For each of the KIBA pairs, each CM (and DTA model) predicted a $K_d$ score for the original $K_s$ value enabling the learning of a CM-specific numerical mapper translating the $K_d$ prediction to it’s $K_s$ counterpart. This has considerable demonstrated impact on the magnitude-specific RMSE results reported in Table 5. For each of the CM models over the validation and test datasets of both the DeepDTA-Defined and Double-Cold datasets, the CM-NM values show a marked improvement (Table 5 where darker values indicate improved performance).

Conversely, when measured using CI, the application of the numerical mapping layer has little to no effect on performance. Since the CI ignores the magnitude of the predicted binding affinity and rather reports the relative ordering of predictions, the remapping of predicted binding affinities to an alternative numerical domain contributes little to the model performance since no additional information has been incorporated (Table 4). However, when the RP layer is applied, which incorporates the context-derived features, a considerable improvement of performance is observed (Table 4).

### Reciprocal perspective improves all component model performance

**Figure 5:**
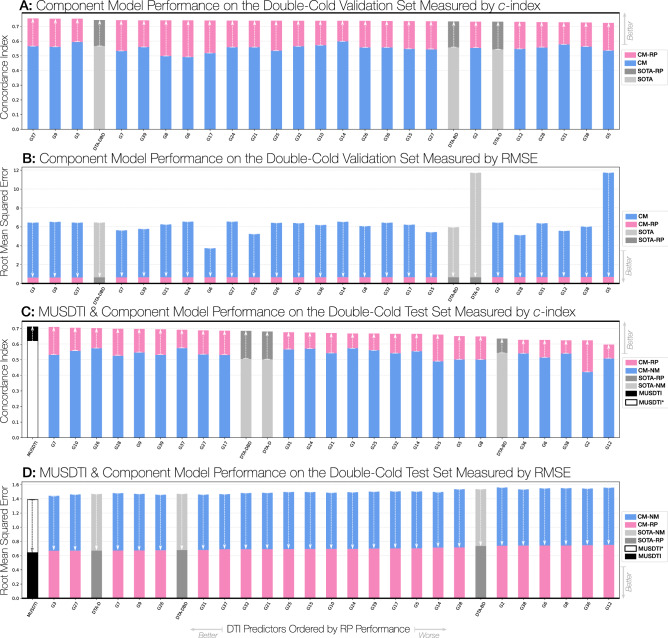
Component model performance improvement from the reciprocal perspective cascaded layer over the double-cold dataset.

**Figure 6:**
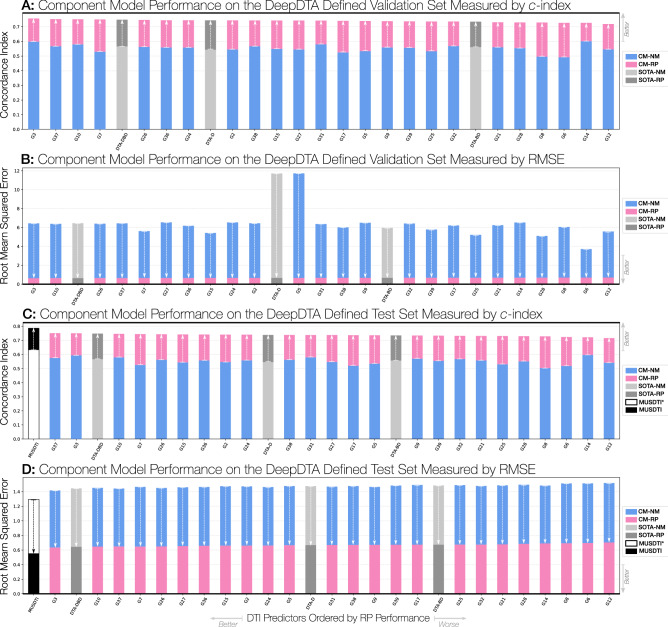
Component model performance improvement from the reciprocal perspective cascaded layer over the DeepDTA-defined dataset.

**Figure 7:**
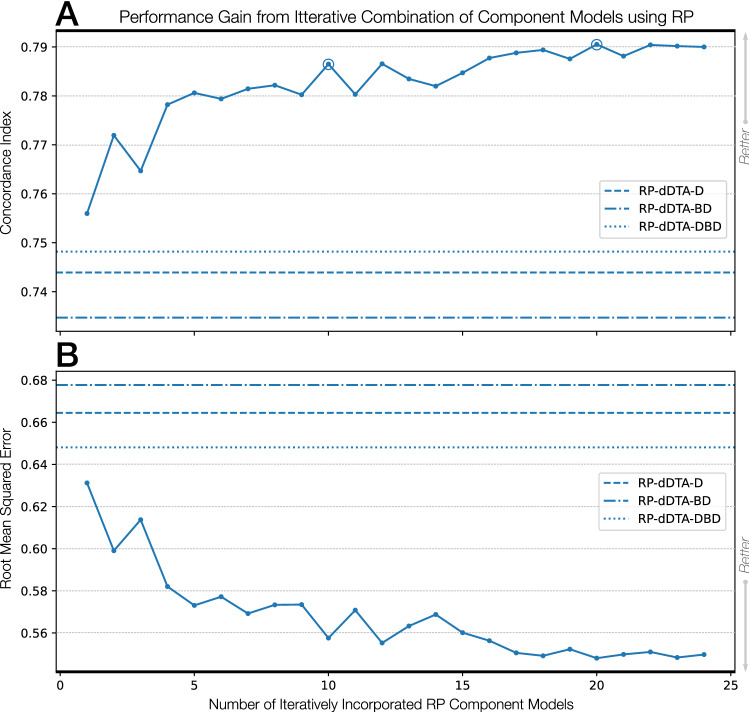
Experimental results over the DeepDTA-defined datasets when incrementally incorporating reciprocal perspective component models compared to the SOTA DeepDTA models. The top-performing combined models were circled in the figure (top-20 models) and the first (top-10 models) represent the performance of the proposed MUSDTI model even when the later combined models represent a marginally higher performance. We opted for the component model ensemble that represented the plateaued performance of component models.

The application of a cascaded RP layer to all student and SOTA models resulted in a considerable improvement in performance. For both the DeepDTA-Defined and Double-Cold validation and test datasets, the CM-RP model results produced a notable increase in performance, whether measured by RMSE or CI (Tables 4, 5; Figs. 5, 6, 7). Most notably, among all reported results, several student-produced models outperform the best ranked DeepDTA model. Interestingly, all models benefit from RP to achieve an approximately similar level of performance, regardless of the metric used. Most importantly, the relative performance between the CM-NM and CM-RP results suggest that the observed performance gain is attributable to the leveraging of context-specific features and not to the domain transfer alone.

Promisingly, these results suggest that any existing SOTA DTI model could benefit from the application of RP as a cascaded layer. These findings corroborate the findings presented in Kyrollos et al. applying RP to miRNA-target prediction where 26 unique SOTA predictors were significantly improved^43^. In an extension of prior work, we sought to not only demonstrate RP as a means of learning a domain transform and to improve performance, but to additionally leverage the CM-specific RP features as part of an ensembled method.

### The MUSDTI model outperforms the state-of-the-art

Prior DTI literature suggests that the incorporation of individual DTI predictors into an ensemble will outperform those individual models^50^. The work of Dick et al. on predicting protein-protein interactions (PPI) between SARS-CoV-2 and humans demonstrated that RP could be used to ensemble two PPI predictors (the Protein-protein Interaction Prediction Engine [PIPE4]^51^ and the Scoring PRotein INTeractions [SPRINT]^52^ models) to produce an RP fusion model^48^.

Here, we sought to expand upon this work to produce a multi-CM DTI ensemble model using Reciprocal Perspective. In the pursuit of the highest-possible performing model, we sought to define the Meta-Undergraduate Student DTI (MUSDTI) model as a meta-model fusing the top-performing CM-RP models. To that end, we incrementally incorporated the derived RP features of each component model (ranked according to their relative performance over the validation dataset) and visualised the performance increase in Fig. 7.

Most excitingly, we note that the inclusion of a single student CM model (without the RP cascade) outperforms the DeepDTA-$*$ and, as expected, the iterative incorporation of CM-RP features results in the progressive performance increase of the ensembled model. The incorporation of the $n=10$ top-ranking student-produced CM models within the RP ensemble appears to have plateaued however we additionally note that the model produced at $n=25$ achieved the maximum recorded performance (Fig. 7). As a trade-off between performance increase (seemingly within random variation at the plateau) and computational expense, we determined that the MUSDTI model would be defined by the ensemble of these top-10 ranking models and all subsequent performance evaluation is made with this model.

The MUSDTI model outperformed not only the student CM and SOTA DeepDTA-$*$ models, but all of their CM-NM and CM-RP variants, supported over the two datasets and performance metrics (Tables 4, 5; Figs. 5, 7). Our findings suggest that ensembled RP-DTI models establish a new SOTA for DTI prediction and provide quality predictions for even the most challenging datasets.

### Defining a double-cold evaluation framework for future DTI studies

In this work, we proposed a novel experimental design that integrates the three most common DTI benchmark datasets, despite their differing DTA measures. This experimental design dedicated two benchmark datasets (Davis and BindingDB) with compatible DTA measures (i.e. $K_d$), which when combined, represent a sizable training dataset that is (by definition) greater than what they represent individually. The third (KIBA) dataset, with $K_s$ values, is then used for the training of a cascaded model by means of transfer learning with the context-leveraging RP framework to further improve performance. This experimental framework that maximally incorporates the three benchmark datasets enabled the definition of a highly conservative double-cold dataset for which none of the pairs contained a drug compound or protein sequence occurring within the training dataset (also known as “Double-Cold”). To our knowledge, this is the first definition of such a conservative test dataset while leveraging the Davis, BindingDB, and KIBA datasets combined in such a cascade to report performance. We recommend that the training and evaluation framework introduced within this work serve as the basis for future DTI work and we further support this initiative by providing the data and component models generated from this work to the research community.

We would like to emphasize that, by definition, the double-cold formulation (where neither the drug SMILES nor protein amino acid sequence in the test set is present in the training or validation sets) is the most conservative experimental design for pairwise model evaluation. In fact, this conservative experimental design was initially proposed for protein-protein interaction prediction tasks in the work of Park and Marcotte^53^. Three levels of difficulty are defined in this critique of evaluation schemes: the easiest has both elements of a test pair appearing in the training dataset, the intermediate has either element of a test pair appearing in the training set, and the hardest has neither of the elements appearing in the training set. The hardest experimental design represents the most authentic evaluation of a model’s performance given that it expresses the model’s expected performance when used to make predictions for completely novel elements (never seen protein amino acid sequences and/or never seen drug SMILES).

To quantitatively express the diversity of the double-cold dataset, we measured the cosine distance between the centroid of the SMILES samples in the training and test set when numerically embedded in their latent space. As expected, the similarity between the double-cold test and training sets with (mean similarity 0.1694 and variance 0.0047) is smaller than the similarity between the DeepDTA-defined test and training sets (mean similarity 0.1711 and variance 0.0714) since higher values are more similar and lower values are more dissimilar. Thus, the double-cold dataset represents an adequately diverse dataset with which to benchmark other DTI prediction methods.

As summarized in Tables 4 and 5, when comparing the performance between the DeepDTA-Defined and Double-Cold datasets, we note that typically the CM-RP validation results outperforms the CM-RP test results (as expected) and that the ensembled MUSDTI model outperformed all models. We note that the CM-NM entry for MUSDTI is the reported result of the ensembled model using only a numerical mapping component and not the full context-leveraging feature set that the RP framework provides.

### Reciprocal perspective enables low computation transfer learning

In this work we demonstrate that domain transfer learning can be easily achieved by learning a CM-NM model to re-score predicted DTI predictions to alternative domains. As expected, the simple non-linear translation from one distribution to another (e.g. $K_d \rightarrow K_s$) while impactful on a magnitude-focused measure, such as RMSE, doesn’t actually incorporate additional learning-specific information, as exemplified in our rank-focused measurement, CI.

However, the application of the context-leveraging Reciprocal Perspective framework not only provides domain translation but also considerably improves the performance of the original model through a downstream cascade. With 14 computed features contributing to this improved model, RP effectively enables a low computation transfer learning layer that may ultimately be used for other domain translation tasks. Excitingly, this work represents the third bioinformatic-related application with demonstrated improvement due to the Reciprocal Perspective framework. Promisingly, the domain-agnostic nature of RP suggests that it may be broadly applied to numerous pair-wise applications, even beyond bioinformatics.

### On the potential for overfitting

An important consideration for the use of the multiple dataset and multiple contributing models is the potential risk of overfitting. In this work, careful steps to de-duplicate datasets were taken to ensure that no replicated samples appeared in multiple independent sets. In fact, the formulation of a super learner training protocol (which relates to stacked generalization in general) avoid the possibility of overfitting by splitting all data into training, validation, and test sets. The CMs are initially created using explicit training data and the input of the downstream meta-model is the out-of-sample predictions. By then training the meta-model on out-of-sample predictions of the CMs, the meta-modal learns how to simultaneously “correct” the out-of-sample predictions and how to best integrate these predictions from multiple CMs to produce its final prediction. Finally, to fully assess the capabilities of the meta-model, it is then evaluated on a final independent test set not used for either the training of the CMs nor the meta-model.

In interpreting the quantitative results of the CM and MUDSTI on the validation and test sets for both the double-cold and DeepDTA-defined sets, we note that test set results are typically lower than the validation set results and within the general range of performance suggesting that these models are not likely to be overfit (Tables 4, 5).

### SHAP analysis reveals variable-contribution CMs and RP features

This work sought to demonstrate the utility of using RP for combining multiple deep learning component models into a single, high-performing MUSDTI model, however, we note that the use of (deep) learning models typically represent black box from which little actionable knowledge may be derived. Thankfully, the machine learning research community is actively engaged in developing explanatory artificial intelligence (XAI) methods that help describe what it is that a particular model focuses upon when generating a given prediction^54^.

One such XAI framework is the SHAPley Additive exPlanations (SHAP) visualization tool that can make a machine learning model more explainable by visualizing the model output^55^. Shapley values are a concept originating from the field of cooperative game theory whose objective is to quantify a given player’s contribution to a game^55^. Shapley values are derived from gameplay contexts where *n* players collectively seek to obtain a reward *p* which is intended for fair distribution among the *n* players according to the individual contribution; such a contribution is known as the *Shapley value*^55^. In the context of XAI, Shapley values are determined through a heuristic game-theoretic framework to quantify the level of contribution a given feature has on a particular model prediction and to determine these contributions on average.

**Figure 8:**
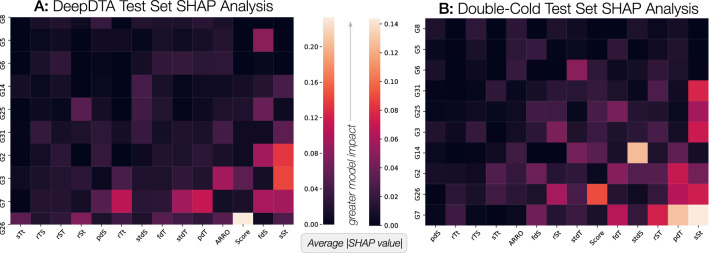
Shapeley additive features analysis. The x-axis is sorted left-to-right in increasing magnitude of SHAP value summed over the column while the y-axis is sorted top-down in increasing magnitude of SHAP value summed over the row. Emanating out from the bottom-right are the models and features with increasingly lesser impact on the model decision. Only the top-10 models contributing to the MUSDTI model are depicted along all 14 RP features.

For the purposes of this work, we are interested in better understanding which of the RP features are most impactful on model performance as well as which of the CMs contributes most to the MUSDTI prediction. Thus, we can conceptualize visualizing the impact of individual features and individual CMs as a matrix/heatmap representing the average SHAP value between that feature and the specific model (Fig. 8). We note that no one model (column-wise) nor RP feature (row-wise) appears to dominate all others and rather, a mixture in the diversity of both the contributing CMs and the features that they provide to support the meta-model output are necessary (Fig. 8). Nonetheless, there are a subset of RP features and component models that appear to contribute more than others. The precise definitions for each feature is listed in Table 1 and the implementation details for each of the CM models are explicitly stated within a Table 1 in the supplementary material. For any high-contributing component model, the specific drug and protein encoding method can be determined from the table and the hyperparameters used for its training are listed. Ultimately, this analysis demonstrates that different models will variably rely on different features to inform the final prediction which is consistent with the utility of ensemble methods that seek to integrate multiple learners.

### Didactics tailored towards resolving real-world problems using limited computational environments

Finally, this work represents an impactful application of senior undergraduate student pedagogy to an open research application. We demonstrated that teams of undergraduate students can, with very limited (and free) cloud-based resources, produce DTI models that rival the state-of-the-art. Most excitingly is the prospect that (under)graduate pedagogy represents a seemingly untapped resource from which advances at the frontier of knowledge may be gleaned. Given the success reported from the MetaStudent work of Dr. Burkhard Rost^31^ and exemplified in the generalized Kaggle framework, there exists ample opportunity to engage (under)graduate students in meaningful ways to advance the state-of-the-art of various applications.

To that end, we strongly encourage researchers and fellow educators to follow from this work and tailor course-specific didactics to promote engagement in projects that may advance the frontier of knowledge. MUSDTI, while benefiting from the application of RP to student-contributed models, could serve in a similar way as a pedagogical example to student- or SOTA-generated models in other application domains. With a well-defined computational framework from which students might explore and evaluate their work, our experiences may be translated to other open research questions.

## Conclusion

The identification of novel DTI is critical to drug discovery and drug repurposing, and represents an open research question for which the research community is actively seeking novel solutions. Various databases contributed to experimentally derived DTI predictors that can be effectively leveraged to achieve SOTA performance, even if different measures of interaction are used in each of the databases.

In this work, we formulated a DTI competition as part of the coursework for an senior undergraduate machine learning course and challenged students to generate component DTI models that might surpass SOTA models and ultimately combined these component models as part of a meta-mode (denoted MUDTI) using the Reciprocal Perspective framework. Consequently, our proposed MUSDTI model represents the new SOTA DTI model.

Our work demonstrated that RP can considerably improve SOTA DTI predictors, that our novel double-cold experimental test dataset (in theory) is better suited to emergent DTI models, that our novel MUSDTI model outperforms SOTA models, that generally, the RP framework can improve individual models as an ensembling method, that RP can be effectively leveraged for the combination of multiple experts (for $n>2$), and that RP can be used in a domain-mapping strategy. Ultimately, we demonstrate that, much like Hamp et al.^31^, student didactics can be tailored to open research applications. Ultimately, this work introduces a novel DTI predictor and revelations for the bioinformatics community in general. This work will share the double-cold test dataset as well as the component models and their domain-transfered predictions of the KIBA dataset to enable future research.

## Supplementary Information

Supplementary Information.

## Data Availability

The materials provided to students in support of this work are available in the following Github repository: https://github.com/jrgreen7/SYSC4906/tree/master/F2020.
