# Supplementary material for: Reciprocal perspective as a super learner improves drug-target interaction prediction (MUSDTI)
Source: Sci Rep. 2022 Aug 2;12:13237. doi: 10.1038/s41598-022-16493-9 (PMC9344797; doi:10.1038/s41598-022-16493-9)
Supplement: Supplementary file 1 — Supplementary Information. [file 41598_2022_16493_MOESM1_ESM.pdf]

Supplementary Materials

MUSDTI: Meta Undergraduate Student Drug-Target Interaction Predictor

Kevin Dick[1,2,*], Daniel G. Kyrollos[1,2], Eric D. Cosoreanu[1,+], Joseph Dooley[1,+], Joshua S. Fryer[1,+], Shaun M. Gordon[1,+], Nikhil Kharbanda[1,+], Martin Klamrowski[1,+], Patrick N. L. LaCasse[1,+], Thomas F. Leung[1,+], Muneeb A. Nasir [1,+], Chang Qiu[1,+], Aisha S. Robinson[1,+], Derek Shao[1,+], Boyan R. Siromahov[1,+], Evening Starlight[1,+], Christophe Tran[1,+], Christopher Wang[1,+], Yu-Kai Yang[1,+], and James R. Green[1,2]

[1]Department of Systems & Computer Engineering, Carleton University, Ottawa, Ontario, Canada
[2]Institute of Data Science, Carleton University, Ottawa, Ontario, Canada
[*]kevin.dick@carleton.ca
[+]these authors contributed equally to this work

ABSTRACT

This document contains all relevant materials to the MUSDTI meta-undergraduate student drug-target interaction predictor. These materials outline all of the (under)graduate student methods to fully describe the MUSDTI model.

Table S1. SOTA, Component, and MUSDTI Model Descriptions and Selected Hyperparameters.

Model	Encodding Method		Libraries Used	Model Architechture/Methods Used	Model Descriptor/Hyperparameters
	Drug SMILE	Protein Amino Acid Sequence			
DTA-*	CNN	CNN	DeepPurpose	Fully-Connected CNN	Model hyperparameters: cls_hidden_dims = [1024,1024,512], train_epoch = 100, LR = 0.001, batch_size = 256, cnn_drug_filters = [32,64,96], cnn_target_filters = [32,64,96], cnn_drug_kernels = [4,6,8], cnn_target_kernels = [4,8,12]
G2	CNN	Transformer	DeepPurpose	Message-Passing Neural Network	Model hyperparemeters: cls_hidden_dims = [1024,1024,512], train_epoch = 100, test_every_X_epoch = 10, LR = 0.001, batch_size = 128, hidden_dim_drug = 128, mpnn_hidden_size = 128, mpnn_depth = 3, transformer_dropout_rate = 0.1, cnn_target_filters = [32,64,96], cnn_target_kernels = [4,8,12]
G3	Two model fusion: Morgan Encoding for the first and CNN for the second	CNN	DeepPurpose	CNN Architecture, Fully-Connected CNN	Configuration Morgan/CNN model config {'LR': 0.001, 'batch_size': 256, 'binary': False, 'cls_hidden_dims': [1024, 1024, 512], 'cnn_target_filters': [32, 64, 96], 'cnn_target_kernels': [4, 8, 12], 'decay': 0, 'drug_encoding': 'Morgan', 'hidden_dim_drug': 256, 'hidden_dim_protein': 256, 'input_dim_drug': 1024, 'input_dim_protein': 8420, 'mlp_hidden_dims_drug': [1024, 256, 64], 'num_workers': 0, 'result_folder': './result/', 'target_encoding': 'CNN', 'train_epoch': 100}. Config CNN/CNN Model {'LR': 0.001, 'batch_size': 256, 'binary': False, 'cls_hidden_dims': [1024, 1024, 512], 'cnn_drug_filters': [32, 64, 96], 'cnn_drug_kernels': [4, 6, 8], 'cnn_target_filters': [32, 64, 96], 'cnn_target_kernels': [4, 8, 12], 'decay': 0, 'drug_encoding': 'CNN', 'hidden_dim_drug': 256, 'hidden_dim_protein': 256, 'input_dim_drug': 1024, 'input_dim_protein': 8420, 'num_workers': 0, 'result_folder': './result/', 'target_encoding': 'CNN', 'train_epoch': 100}
G5	CNN_RNN	Transformer	DeepPurpose	Transformer Architechture	Model Hyperparameters: cls_hidden_dims = [1024,1024,512], cnn_drug_filters = [32,64,96], cnn_drug_kernels = [4,6,8], rnn_Use_GRU_LSTM_drug = 'GRU', rnn_drug_hid_dim = 64, rnn_drug_n_layers = 2, rnn_drug_bidirectional = True, transformer_emb_size_target = 128, transformer_intermediate_size_target = 256, transformer_num_attention_heads_target = 4, transformer_n_layer_target = 2, transformer_dropout_rate = 0.20, transformer_attention_probs_dropout = 0.2, transformer_hidden_dropout_rate = 0.2, LR = 0.001, train_epoch = 20, batch_size=64
G6	Pubchem	PseudoAAC	Keras	Multi-Layer Perceptron	Model Hyperparameters: hidden layer sizes - [196, 128,64] with 40% dropout layers between each, trained for 30 epochs, batch size of 512, optimizer=RMSprop(lr=0.001).
G7	CNN	CNN	DeepPurpose	Fully-Connected CNN Architecture, Hyperparameter Tuning	The model hyperparameter used are as follows: cls_hidden_dims = [2024,2024,1024], epoch = 100, learning rate = 0.001, batch_size = 256, cnn_drug_filters = [32,64,96, cnn_target_filters = [32,64,96, cnn_drug_kernels = [4,6,8], cnn_target_kernels = [4,8,12] .
G8	CNN_RNN	CNN_RNN	DeepPurpose	Fully-Connected CNN Architecture, Hyperparameter Tuning	The model hyperparameter used are as follows: cls_hidden_dims = [1024,1024,512], train_epoch = 25, LR = 0.0001530038096010685, batch_size=256, cnn_drug_filters = [32,64,96], cnn_target_filters = [32,64,96], cnn_drug_kernels = [4,6,8], cnn_target_kernels = [4,6,8], rnn_drug_n_layers = 64, rnn_target_n_layers = 32, hidden_dim_drug=128, hidden_dim_protein=64, rnn_drug_hid_dim = 3, rnn_target_hid_dim = 2
G9	Morgan	Conjoint_triad	DeepPurpose	Fully-Connected CNN Architecture, Hyperparameter Tuning	The model hyperparameter used are as follows: cls_hidden_dims = [1024,1024,512], train_epoch = 500, LR = 0.0001, batch_size = 128, hidden_dim_drug = 128, mpnn_hidden_size = 128, mpnn_depth = 3, cnn_target_filters = [16,32,64,96,128], cnn_target_kernels = [2,4,8,12,24]
G10	MPNN	CNN	DeepPurpose	Fully-Connected CNN Architecture, Hyperparameter Tuning	The model hyperparameter used are as follows: cls_hidden_dims = [1250,1250,700], train_epoch = 75, LR = 0.001, batch_size = 256, cnn_drug_filters = [40,60,80], cnn_target_filters = [40,60,80], cnn_drug_kernels = [4,6,8], cnn_target_kernels = [4,6,8]
G12	CNN	CNN	DeepPurpose	Fully-Connected CNN Architecture, Hyperparameter Tuning	The model hyperparameter used are as follows: cls_hidden_dims = [512,512,256], train_epoch = 100, LR = 0.001, batch_size = 100, cnn_drug_filters = [32,64,96], cnn_target_filters = [32,64,96], cnn_drug_kernels = [4,6,8], cnn_target_kernels = [4,8,12]
G14	CNN	CNN	Keras	Fully-Connected CNN Architecture, Hyperparameter Tuning	The final model hyperparameters were determined following 50 iterations of tuning. Model hyperparameters: cls_hidden_dims = [1024,1024,512], train_epoch = 100, LR = 0.001, batch_size = 128, cnn_drug_filters = [32,64,96], cnn_target_filters = [32,64,96], cnn_drug_kernels = [4,6,8], cnn_target_kernels = [4,8,12]
G15	Transformer Encoder on ESPF	Transformer Encoder on ESPF	DeepPurpose	Feed-Forward Neural Network/(deep) Multi-Layer Perceptron/(deep) Artificial Neural Network, Transformer Network, Dropout Layers used	This model is a modified version of MT-DTI that uses the Transformers encodings. It sacrifices representational capability (model depth) for greater target context (model width). hyperparameters = {transformer_emb_size_drug: 128, transformer_intermediate_size_drug: 512, transformer_num_attention_heads_drug: 4, transformer_n_layer_drug: 2, transformer_emb_size_target: 128, transformer_intermediate_size_target: 516, transformer_num_attention_heads_target: 4, transformer_n_layer_target: 2, transformer_dropout_rate: 0.1, transformer_attention_probs_dropout: 0.1, transformer_hidden_dropout_rate: 0.1}
G17	CNN_RNN	CNN_RNN	DeepPurpose	Fully-Connected CNN Architecture, Hyperparameter Tuning	Model hyperparameters: cls_hidden_dims = [1024,1024,512], train_epoch = 20, LR = 0.0005, batch_size = 256, cnn_drug_filters = [32,64,96], cnn_target_filters = [32,64,96], cnn_drug_kernels = [4,6,8], cnn_target_kernels = [4,8,12], rnn_Use_GRU_LSTM_drug = 'GRU', rnn_drug_hid_dim = 64, rnn_drug_n_layers = 2, rnn_drug_bidirectional = True, rnn_Use_GRU_LSTM_target = 'GRU', rnn_target_hid_dim = 64, rnn_target_n_layers = 2, rnn_target_bidirectional = True
G21	CNN_RNN	CNN_RNN	PyTorch	Multi-Layer Perceptron	Hidden layer dimensons[1024,1024,512] drug filters[32,64,96] target filter [32,64,96] drug kernel [4,6,8] target kernel [4,8,12] learning rate 0.001 training epochs 100
G24	CNN	Transformer	DeepPurpose	Fully-Connected CNN Architecture, Hyperparameter Tuning	Model hyperparameters: cls_hidden_dims = [1024,1024,512], train_epoch = 350, batch_size = 256, cnn_drug_filters = [32,64,96], cnn_target_filters = [32,64,96], cnn_drug_kernels = [4,6,8], cnn_target_kernels = [4,8,12]
G25	CNN	CNN_RNN	TensorFlow, Keras, DeepPurpose	CNN Architecture, ResNet Fine-Tuning/Transfer-Learning, Transformer Network	Cons rebates results are fed into a ResNet model. The drug sequence is encodded using a CNN, the protein sequence is encoded through an LSTM, and finally the layers are concatenated.<br>Concatenated result are fed into two ResNet-like residual layers that then leads into a fully-connected layer to produce the prediction.
G26	CNN	CNN	Keras, Scikit-Learn, TensorFlow	Fully-Connected CNN	The model consists of a convolution, max-pooling, fully-connected and, finally an output sigmoid layer. Model hyperparamenters: cls_hidden_dims = [1024,1024,512,512], cnn_drug_filters = [32,64,96], cnn_target_filters = [32,64,96], cnn_drug_kernels = [4,6,8], cnn_target_kernels = [4,8,12], LR = 0.00135, train_epoch = 85, batch_size=256
G27	CNN	CNN	Keras, TensorFlow	CNN Architecture, Fully-Connected CNN, GRU Network, Dropout Layers used	DeepDTA-based model that outputs into a sequential pair of 192-unit Bi-GRU layers. Too many model parameterizations to include in this table: precise model architechture values are available upon request.
G28	CNN	CNN_RNN	DeepPurpose	Fully-Connected CNN Architecture, Hyperparameter Tuning	Model hyperparameters: hidden dimensions of classifier = [1024,1024,512], training epoch= 10, LR = 0.001, Cnn drug filters = [32,64,96], Cnn drug kernels = [4,6,8], Cnn target filters = [32,64,96], Cnn target kernels = [4,8,12], Rnn target hidden dimensions = 64, Rnn target n layers = 3
G31	CNN_RNN	CNN_RNN	Scikit-Learn, DeepPurpose	Fully-Connected CNN Architecture, Hyperparameter Tuning	Model Hyperparameters: cls_hidden_dims = [1024,1024,512], train_epoch = 100, LR = 0.001, batch_size = 256, hidden_dim_drug = 64, cnn_drug_filters = [32,64,96], cnn_target_filters = [32,64,96], cnn_drug_kernels = [4,6,8], cnn_target_kernels = [4,8,12], rnn_drug_n_layers = 4
G32	CNN	CNN	DeepPurpose	Fully-Connected CNN Architecture, Hyperparameter Tuning	Model Hyperparameters: cls_hidden_dims = cls_hidden_dims = [1024, 1024, 512], cnn_drug_filters = [32, 64, 96], cnn_target_filters = [32,64,96], cnn_drug_kernels = [4,6,8], cnn_target_kernels = [4,8,12], LR = 0.0001, train_epoch = 50
G36	CNN	CNN	DeepPurpose	Fully-Connected CNN Architecture, Hyperparameter Tuning, SPVec Experimentation	Model Hyperparameters: cls_hidden_dims = [1024,1024,512], train_epoch = 100, LR = 0.001, batch_size = 128, cnn_target_filters = [32, 64, 96], cnn_target_kernels = [4, 8, 12], cnn_drug_filters = [32, 64, 96], cnn_drug_kernels = [4, 6, 8]
G37	Morgan	Conjoint_triad	DeepPurpose	Fully-Connected CNN Architecture, Hyperparameter Tuning	Model Hyperparameters: cls_hidden_dims = [1024,1024,512], train_epoch = 200, LR = 0.001, batch_size = 128,hidden_dim_drug = 128, mpnn_hidden_size = 128, mpnn_depth = 3
G38	CNN	CNN	DeepPurpose	Fully-Connected CNN Architecture, Hyperparameter Tuning	Model Hyperparameters: cls_hidden_dims = [1024,1024,512], train_epoch = 5, LR = 0.001, batch_size = 128, cnn_target_filters = [32,64,96], cnn_target_kernels = [4,8,12], cnn_drug_filters = [32,64,96], cnn_drug_kernels = [4,6,8]
G39	CNN	CNN	DeepPurpose, Scikit-Learn, PyTorch	CNN Model Architecture	This model leverages a prototypical CNN encoding and model architecture. Model hyperparameters: cls_hidden_dims = [1024,1024,512], train_epoch = 120, LR = 0.001, batch_size = 1024, cnn_drug_filters = [32,64,96], cnn_target_filters = [32,64,96], cnn_drug_kernels = [4,6,8], cnn_target_kernels = [4,8,12]
